# The FBW7-MCL-1 axis is key in M1 and M2 macrophage-related colon cancer cell progression: validating the immunotherapeutic value of targeting PI3Kγ

**DOI:** 10.1038/s12276-020-0436-7

**Published:** 2020-05-22

**Authors:** Yeo Song Lee, Su Jeong Song, Hye Kyung Hong, Bo Young Oh, Woo Yong Lee, Yong Beom Cho

**Affiliations:** 1 Sungkyunkwan University School of Medicine, Seoul, Republic of Korea; 2 Institute for Future Medicine Samsung Medical Center, Seoul, Republic of Korea; 3 Department of Colorectal Surgery, Hallym University Sacred Heart Hospital, Hallym University College of Medicine, Anyang, Korea; 4 Department of Surgery, Samsung Medical Center, Sungkyunkwan University School of Medicine, Seoul, Republic of Korea; 5 Department of Health Sciences and Technology, Samsung Advanced Institute for Health Sciences and Technology, Sungkyunkwan University, Seoul, Republic of Korea

**Keywords:** Cancer microenvironment, Translational research, Immunosuppression

## Abstract

Colorectal cancer is a devastating disease with a low 5-year survival rate. Recently, many researchers have studied the mechanisms of tumor progression related to the tumor microenvironment. Here, we addressed the prognostic value of tumor-associated macrophages (TAMs) using a total of 232 CRC patient tissue samples and investigated the mechanisms underlying TAM-related colon cancer progression with respect to PI3Kγ regulation using in vitro, in vivo, and ex vivo approaches. Patients with M2/M1 < 3 had significantly improved progression-free survival and overall survival compared with patients with M2/M1 > 3. M1 and M2 macrophages elicited opposite effects on colon cancer progression via the FBW7-MCL-1 axis. Blocking macrophage PI3Kγ had cytotoxic effects on colon cancer cells and inhibited epithelial–mesenchymal transition features by regulating the FBW7-MCL-1 axis. The results of this study suggest that macrophage PI3Kγ may be a promising target for immunotherapy in colon cancer.

## Cancer treatment: turning the tide against colon cancer

Drugs that target a specific subset of immune cells could render colorectal tumors more susceptible to immunological destruction by the host. The cellular composition of a tumor profoundly affects the odds of progression or survival, and some immune cell types can stall the antitumor response rather than strengthening it. Researchers led by Yong Beom Cho of Sungkyunkwan University, Seoul, South Korea, explored the impact of various subpopulations of macrophages, cells that help coordinate the immune counterattack against cancer. The researchers learned that the relative balance between M2 and M1 subtypes of macrophages correlates with colorectal cancer outcomes, patients with less M2 and more M1 activity generally faring better. They also uncovered a strategy for inhibiting M2 activity, which unleashes a more-aggressive response against the tumor and could thus offer a useful therapeutic approach.

## Introduction

Colorectal cancer (CRC) is one of the most common malignancies worldwide. To improve the survival rates of patients with CRC, many researchers have studied the underlying mechanisms of carcinogenesis related to both the tumor cell itself and the tumor microenvironment. Like most other solid carcinomas, CRC has a microenvironment that is infiltrated by a variety of immune cells, including tumor-associated macrophages (TAMs), monocytes, dendritic cells, natural killer cells, CD4^+^ T cells, and CD8^+^ T cells^1–3^. Among these immune cells, TAMs are usually the most abundant immune cells in the tumor microenvironment^4–6^. Recruited macrophages adapt to their environment by developing one of two major polarization phenotypes: M1 (classical) and M2 (alternative). TAMs usually have M2 characteristics associated with poor survival in patients with breast cancer^7^, lung cancer^8^, bladder cancer^9^, and ovarian cancer^10^. In contrast, increased TAMs are positively correlated with favorable outcomes in lung cancer^11^, prostate cancer^12^, and stomach cancer^13^. Furthermore, several studies on CRC cases have shown that TAMs can lead to a favorable prognosis^14–19^. In addition, high macrophage infiltration into the invasive front of colon cancer is correlated with low liver metastasis^20^. Hence, continued investigation on the role of TAMs in CRC progression might lead to improved therapeutic strategies. Class I phoaphoinositide 3-kinase (PI3K) signaling regulates metabolic pathways during inflammation and carcinogenesis^21^. PI3Kγ is a class IB isoform of PI3K that is abundantly expressed in myeloid cells, including macrophages. However, PI3Kγ is not expressed in cancer cells^22^. Previous studies have implicated PI3Kγ in the regulation of the innate immune response during cancer progression through TSC, PDK1, and Akt1^23–26^. Furthermore, inhibition of macrophage PI3Kγ downregulates pancreatic ductal adenocarcinoma tumor progression and increases survival rates by altering macrophage transcriptional profiles^27^. As shown above, the roles of M1 and M2 TAMs in CRC and their prognostic value remain ambiguous. In addition, the mechanisms underlying TAM-related cancer progression with respect to PI3Kγ regulation are currently unclear.

Therefore, the objectives of this study were (1) to investigate the correlation between TAM subtype and colon cancer cell progression; (2) to determine the mechanism involved in their interaction; (3) to explore the mechanisms underlying TAM-related cancer progression with respect to PI3Kγ regulation; and (4) to determine the prognostic value of TAM subtype in the CRC microenvironment.

## Materials and methods

### Patients and data collection

From 2006 to 2007, paraffin-embedded samples of CRC patients were obtained from the Department of Surgery, Samsung Medical Center (Sungkyunkwan University School of Medicine), Seoul, Korea. All patients underwent a surgical procedure for colon or rectal cancer, and their data were prospectively entered into a database. Clinical data were retrospectively collected and analyzed based on medical records and operative notes. This study was approved by the Institutional Review Board (IRB) of Samsung Medical Center (IRB No. 2010-09-017). A total of 232 CRC patients (stage I, *n* = 32; stage II, *n* = 70; stage III, *n* = 76; and stage IV, *n* = 54) were analyzed for clinical outcomes, including progression-free survival (PFS) and overall survival (OS).

### Multiplex tissue microarray (TMA) analysis

Tissue cores (2 mm in diameter) were carefully transferred to recipient paraffin blocks. Filled recipient blocks were embedded in paraffin and sectioned (4 µm in thickness). TMA slides were serially stained using the Opal Kit (PerkinElmer, Hopkinton, MA, USA) for immunohistochemical (IHC) analysis. In brief, sections were dewaxed by heating at 55 °C for 30 min followed by three washes with xylene (5 min per wash). The slides were then rehydrated in serial ethanol solutions (100, 90, and 80%) and distilled water (5 min each). Antigen retrieval was achieved using a microwave oven at 100% power followed by 10% power in citrate antigen retrieval buffer (pH 6.0) for 15 min. Endogenous peroxidase activity was blocked by incubating the slides in 0.3% hydrogen peroxide at room temperature for 10 min. Background reactivity was removed using universal blocking serum (Dako Diagnostics, Glostrup, Denmark) for 30 min at room temperature. Slides were then incubated with antibodies specific to CD86 (#ab53004, Abcam, Cambridge, MA) and CD163 (#ab17051, Abcam, Cambridge, MA) for 1 h at room temperature. After primary antibody incubation, the sections were washed with 0.02% Tris-buffered saline containing 0.1% Tween 20 (TBST) and incubated with secondary horseradish peroxidase-conjugated antibodies at room temperature for 10 min. The sections were washed three times with TBST, followed by tyramide signal amplification (TSA) using TSA-Cy3 (PerkinElmer, Boston, MA) and TSA-FITC (PerkinElmer, Boston, MA) at room temperature for 10 min according to the manufacturer’s instructions. All sections were washed five times with TBST (5 min each), followed by DAPI staining (PerkinElmer, Boston, MA). The sections were mounted with Vectashield HardSet medium (Vector, Burlingame, USA). Dried slides were scanned using the PerkinElmer Vectra 3.0 platform at ×20 magnification. Images were subjected to phenotyping and analyzed using inForm Advanced Image Analysis Software (PerkinElmer, Boston, MA). Cells that were positive for CD86 and CD163 were classified as M1 and M2 macrophages, respectively. Cell quantification was performed using Spotfire (TIBCO, Boston, MA).

### Cell cultures, macrophage polarization, and reagents

Human CRC cell lines (HCT116, HT29, LoVo, and SW48), a human monocyte cell line (THP-1), and a mouse colon cancer cell line (CT26) were obtained from American Type Culture Collection (ATCC, Manassas, VA, USA). Cells were cultured in Rosewell Park Memorial Institute (RPMI) 1640 (Gibco, Grand Island, NY, USA) supplemented with 10% fetal bovine serum (FBS, Gibco) and 1% penicillin–streptomycin (Gibco, NY,USA) in a 5% CO_2_ incubator at 37 °C. According to previous studies^28,29^, to polarize M0 macrophages, THP-1 cells (5 × 10^6^) were plated in 100-phi dishes and stimulated with 320 nm phorbol 12-myristate 13-acetate (PMA; Sigma, St. Louis, MO, USA), followed by incubation at 37 °C for 24 h. The culture supernatant was collected and labeled M0 macrophage-conditioned medium (M0 CM). To polarize M1 or M2 macrophages, THP-1 cells were treated with PMA for 6 h, and M1-polarizing reagents (100 ng/ml lipopolysaccharide (LPS) plus 20 ng/ml interferon gamma (IFN-γ); R&D, Waltham, MA) or M2-polarizing reagents (20 ng/ml IL-4 plus 20 ng/ml IL-13) were added, followed by incubation at 37 °C for 18 h. The culture supernatant was collected and labeled M1 macrophage CM (M1 CM) or M2 macrophage CM (M2 CM). MG132 (proteasome inhibitor) and PI3Kγ inhibitors (TG100-115 and IPI-549) were purchased from Sigma-Aldrich (St. Louis, MO, USA) and Selleckchem (Houston, TX, USA), respectively.

### Cell proliferation assay

The cell proliferation assay was performed in triplicate. A colorimetric assay was performed to determine cell viability by evaluating the metabolic conversion of water-soluble tetrazolium salt (WST)-1 (2-(4-iodophenyl)-3-(4-nitrophenyl)-5-(2,4-disulfophenyl)-2H-tetrazolium)) purchased from Roche (Indianapolis, IN, USA). In brief, WST-1 was added directly into culture wells and incubated at 37 °C for 60–120 min. Absorbance was measured at a wavelength of 450 nm. Three different experiments were conducted for each experimental condition.

### Cell lysis and western blot analysis

To obtain whole-cell extracts, cells were lysed with Pro-prep buffer (Intron Biotechnology, Seoul, Korea) containing protease inhibitors. Protein extracts (10–60 μg) were resolved by sodium dodecyl sulfate polyacrylamide gel electrophoresis and transferred to polyvinylidene difluoride membranes. These membranes were probed with SURVIVIN (#AF886, R&D Systems, Waltham, MA), BMI-1 (#sc-10745, Santa Cruz, CA, USA), Caspase-3 (#9662, Cell Signaling, MA, USA), PARP (#9542, Cell Signaling, MA, USA), E-cadherin (#610181, BD Bioscience, San Jose, CA), N-cadherin (#4061, Cell Signaling, MA, USA), VIMENTIN (#sc-32322, Santa Cruz, CA, USA), MMP2 (#13132, Cell Signaling, MA, USA), phospho AKT (#4060, Cell Signaling, MA, USA), AKT (#4691, Cell Signaling, MA, USA), phospho ERK (#612358, BD Bioscience, San Jose, CA), ERK (#9102, Cell Signaling, MA, USA), MCL-1 (#4572, Cell Signaling, MA, USA), FBW7 (#ab10752, Abcam, Cambridge, MA), and β-actin (#3700, Cell Signaling, MA, USA) primary antibodies, followed by incubation with secondary antibodies conjugated to horseradish peroxidase (Santa Cruz Biotechnology, CA, USA). β-actin was used as a loading control for western blot analysis.

### Animals

Six- to seven-week-old female BALB/c nu/nu and BALB/c wild-type mice weighing 15–17 g at the time of transplantation were used in this study. These mice were obtained from Orient Bio (Seoul, Korea) and maintained under specific pathogen-free conditions. This study was reviewed and approved by the Institutional Animal Care and Use Committee (IACUC) of Samsung Biomedical Research Institute (SBRI). SBRI is a facility that is accredited by the Association for Assessment and Accreditation of Laboratory Animal Care International (AAALAC International). This study abided by the Institute of Laboratory Animal Resources (ILAR) guidelines.

### Coculture of polarized macrophages and mouse xenografts

Polarized M0, M1, and M2 macrophages (4 × 10^5^ cells) were seeded into the lower compartment of a six-well Boyden chamber. HCT116 cells (2.5 × 10^5^ cells) were added into the upper inserts. The cells were cocultured at 37 °C for 7 days, followed by 3 days of CM treatment. Tumorigenesis was measured using a mouse xenograft model to evaluate the effects of polarized macrophages on cancer growth. In brief, cocultured HCT116 cells were suspended in PBS supplemented with 50% Matrigel and injected subcutaneously into the flanks of six- to seven-week-old female BALB/c nu/nu mice. Tumor size was measured twice a week with a caliper. Tumor volume was calculated using the following formula: tumor volume = (short length × long length × width)/2. The mice were sacrificed three weeks after transplantation/injection.

### RT-PCR and real-time PCR

Total RNA was extracted using TRIzol reagent (Invitrogen, Carlsbad, CA, USA). Total RNA (500 ng) was then subjected to reverse transcription using a Superscript II reverse transcriptase kit (Invitrogen, Carlsbad, CA, USA) according to the manufacturer’s instructions. Primer sequences used for RT-PCR and real-time PCR are listed in Supplementary Table 1. The mRNA level of each gene was normalized to that of GAPDH or β-actin.

### IHC staining

IHC analysis for Ki67 (#sc-15402, Santa Cruz, CA, USA) was performed on 4-µm frozen sections of obtained mouse xenograft tissues. The tissue sections were deparaffinized with xylene, hydrated in serial dilutions of alcohol, and immersed in 3% H_2_O_2_. Following antigen retrieval in citrate buffer (pH 6.0), the tissue sections were incubated with protein-blocking agent (Immunotech, Marseille, France) at room temperature for 10 min to block nonspecific antibody binding. The sections were then incubated with primary antibody against Ki67 (1:200) in a humidified chamber overnight at 4 °C. After being washed with PBS three times, these sections were incubated with a biotinylated secondary antibody and streptavidin conjugated to horseradish peroxidase (Immunotech, Marseille, France) for 60 min at room temperature, followed by a PBS wash. Chromogen was developed with liquid 3,30-diaminobenzidine (Immunotech, Marseille, France), followed by counterstaining with Meyer’s hematoxylin. The slides were examined under a light microscope.

### Cell invasion and wound-healing assay

Cell invasion assays were performed using uncoated transwell migration chambers (BD Bioscience, San Jose, CA, USA) in 24-well cell culture plates. Cells (5 × 10^4^/well) were loaded into the invasion chamber in serum-free RPMI. The lower chamber contained RPMI supplemented with 10% FBS as a chemoattractant. The plates were incubated at 37 °C for 24 h, followed by staining with hematoxylin–eosin and mounting. Cell invasion was imaged using an upright microscope (×200). The wound-healing assay was performed using l-Dish 35-mm-high culture inserts (Ibidi, Martinsried, Germany) according to the manufacturer’s protocols. In brief, on the day before experimentation, the cells were seeded into each well of the culture inserts and incubated at 37 °C in a humidified atmosphere with 5% CO_2_. After cell attachment, the culture inserts were gently removed using tweezers, and the cells were incubated in macrophage CM) for more than 12 h.

### Immunofluorescence microscopy

After incubation with macrophage CM, HT29 cells were washed twice with cold PBS, fixed in methanol at 4 °C for 10 min, washed with PBS, permeabilized with 0.1% Triton X-100/PBS for 30 min, and incubated with a primary antibody against E-cadherin in 1% BSA/0.1% Triton X-100/PBS at 4 °C overnight. After being washed with PBS, immunolabeled proteins were visualized by treatment with fluorescent-conjugated secondary antibodies for 60 min at room temperature. The cells were washed with PBS, mounted with DAPI mounting medium, sealed with cover slips, and examined using a confocal laser scanning microscope (LSM 780; Carl Zeiss, NY, USA).

### Patient-derived cell (PDC) isolation and dissociation

Surgically resected colon cancer tissues were obtained from patients from Samsung Medical Center (SMC), Seoul, Korea. This study was approved by the Institutional Review Board (IRB) of SMC (IRB No. 20**-0*-017). Tissues were washed three times with 70% ethanol, followed by cold PBS washes until the supernatant was clear. Next, the tissues were chopped into ~5-mm pieces and further washed with cold PBS. These pieces were then incubated with digestion buffer (Dulbecco’s modified Eagle medium with 2.5% FBS, 1% penicillin/streptomycin (Invitrogen, Carlsbad), 75 U/mL collagenase type IV (Gibco, Grand Island, NY), and 125 µg/mL dispase type II (Gibco, Grand Island, NY)) at 37 °C for 30–60 min. Digested cells were then centrifuged at 200 g for 3 min to separate adenoma from single cells. Dissociated cells were passed through a 40-µm cell strainer and washed several times with PBS. Isolated colon cancer cells were counted and embedded in Matrigel on ice and seeded into 24-well cell culture plates.

### Flow cytometry and morphology analysis

Cells isolated from mouse tumors were incubated with anti-CD16/32 antibody (Fc blocker, #553142, BD, San Jose, CA) to block nonspecific binding and were stained with the following fluorochrome-tagged antibodies: anti-CD11b APC-Cy7 (#557657, BD, San Jose, CA), anti-CD11b APC-Cy7 isotype control (# 400623, Biolegend), anti-F4/80 Alexa 647 (#565853, BD, San Jose, CA), anti-F4/80 Alexa 647 isotype control (#557690, BD, San Jose, CA), anti-MHC Class II eFluor 450 (#48–5321–82, Invitrogen, Carlsbad, CA, USA), anti-MHC Class II eFluor 450 isotype control (#48-4031-80, Invitrogen, Carlsbad, CA, USA), anti-CD206 PE (#12-2061-82, Invitrogen, Carlsbad, CA, USA), and anti-CD206 PE isotype control (#12-4031-81, Invitrogen, Carlsbad, CA, USA) antibodies. Stained cells were then analyzed with a Verse flow cytometer and BD FACS Diva software (BD Biosciences, San Jose, CA). Dead cells and doublets were excluded based on forward scatter and side scatter.

### Statistical analysis

Data obtained from xenograft models were analyzed using GraphPad Prism 5.0 software (La Jolla, CA, USA) with one-way analysis of variance (ANOVA) and post hoc analysis (Bonferroni post hoc test). For clinical data analysis, statistical processing was conducted using SPSS version 19.0 software (SPSS Inc., Chicago, IL, USA). Survival rates were estimated using the Kaplan–Meier method and compared by the log-rank test. The data are presented as the mean ± SD. Statistical significance was considered at *p* < 0.05.

## Results

### Differentiation of macrophages into particular subtypes

THP-1 cells were differentiated according to classical conditions (PMA to polarize macrophages and LPS plus IFN-γ or IL-4 plus IL-13 to differentiate M1 or M2 macrophages, respectively). THP-1 cells showed a round and partly clustered morphology and grew in suspension. During the polarization process, THP-1 cells underwent morphological changes, including circle-like shape attachment, size increases in M0 and M2 macrophages, and fibroblast-like changes in M1 macrophages (Supplementary Fig. 1A). RT-PCR analysis showed that *CCR7* and *CXCL9* (two major M1 macrophage markers) were exclusively expressed in M1-differentiated macrophages, whereas *CCL17* and *CD23* (well-known M2 macrophage markers) were significantly expressed in M2-differentiated macrophages (Supplementary Fig. 1B). To further delineate macrophage subtypes, the expression levels of cytokines that are generally responsible for proinflammatory and antiinflammatory responses in polarized macrophage subtypes were evaluated by RT-PCR. Proinflammatory cytokines such as *IL-1α*, *IL-1β*, *CXCL10*, *IL-8*, and *IL-12β* were upregulated in M1 macrophages compared with M2 macrophages (Supplementary Fig. 1C, upper panel). However, antiinflammatory cytokines such as *TGF-β* and *IL-10* were upregulated in M2 macrophages compared with M1 macrophages (Supplementary Fig. 1C, lower panel).

### M1 macrophage CM impedes colon cancer cell viability through apoptosis

**Fig. 1:**
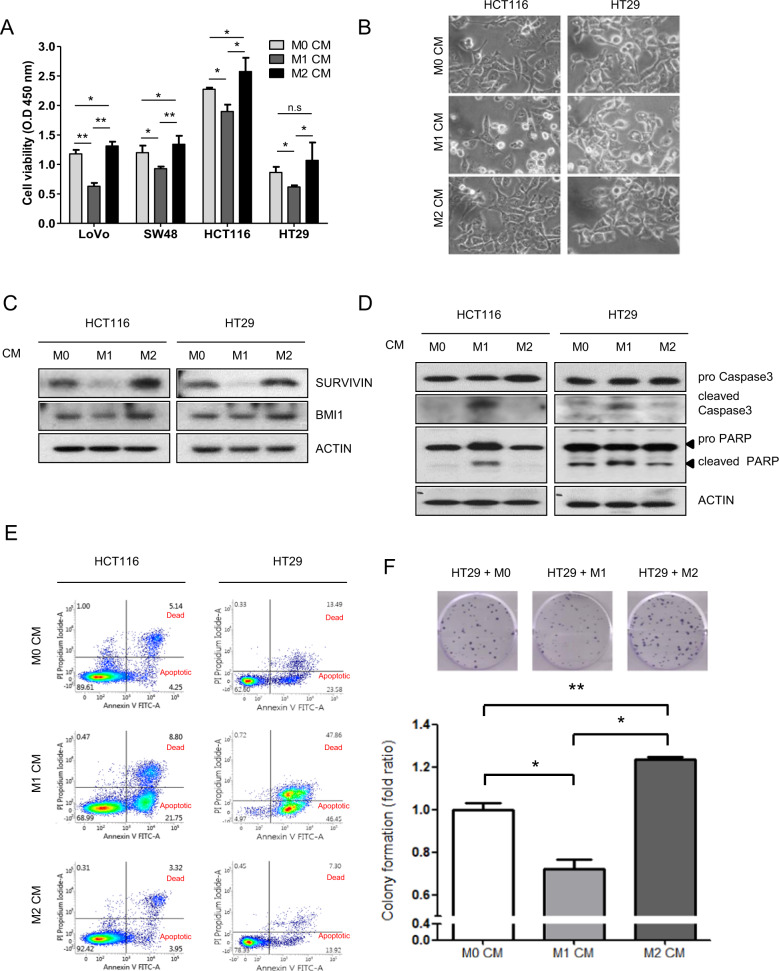
Opposite effects of M1 and M2 macrophages on the viability of colon cancer cells. **a** Viability of four colon cancer cell lines after coculture with macrophage CM for 24 h based on the WST-1 assay. Error bars are derived from three independent experiments. **b** Phase contrast microscopy observation (×400) of HCT116 and HT29 cells treated with macrophage CM for 24 h. **c** Expression levels of SURVIVIN and BMI-1 in macrophage CM-treated HCT116 and HT29 cells based on western blotting. **d** Extent of caspase-3 activation and cleavage of PARP in cell lysates, as measured by western blotting. **e** Apoptotic cells were determined by FACS using annexin V/PI double staining after 24 h of incubation with each macrophage CM with HCT116 and HT29 cells. **f** The colony formation effect of each macrophage CM on HT29 cells. Representative images of the colony-forming assay (upper panel) and analysis of colony formation rates (lower panel) are shown. The results are presented as the mean value ± SE. *P < 0.05; **P < 0.01; ***P < 0.001.

Conditioned media (CM) from differentiated macrophages was collected after 24 h of polarization, and the effect of CM on viability of normal colon cell (CCD-18Co, Supplementary Fig. 1D) and colon cancer cell were investigated. Coculture with M1 CM clearly decreased the viability of LoVo, SW48, HCT116, and HT29 cells. However, M2 CM slightly increased the viability of these colon cancer cells compared with that of M0 CM (Fig. 1a). Among these colon cancer cells, the HCT116 and HT29 cell lines were chosen for further study. As hypothesized, our results revealed that M1 CM had different effects on morphological changes in both HCT116 and HT29 cells (Fig. 1b). The morphology of M1 CM-treated cells changed from a spindle-like to a pebble-like shape. In addition, the cell-to-cell contact of M1 CM-treated cells became less dense than that of M0 CM-treated cells. Furthermore, vacuole formation was significantly increased in M1 CM-treated cells compared with that of M0 CM-treated cells. M1 CM-treated cells also showed morphological characteristics of apoptosis. On the other hand, M0 or M2 CM-treated HCT116 and HT29 cells did not show apoptotic features. Furthermore, the proliferation of M2 CM-treated cells was increased compared with that of M0 CM-treated cells. Therefore, apoptosis-related cell death was examined based on apoptosis markers using western blotting and RT-PCR analysis. Western blotting results showed that the expression levels of the antiapoptotic markers SURVIVIN and BMI-1 were attenuated in M1 CM-treated cells. However, M2 CM increased the expression levels of these markers in HCT116 and HT29 cells (Fig. 1c). The results of RT-PCR analysis showed the same expression pattern for *SURVIVIN* in CM-treated HCT116 and HT29 cells. The expression of *p21*, a well-known apoptosis marker, was increased in M1 CM-treated cells. However, M2 CM treatment had the opposite effect (Supplementary Fig. 2A). SURVIVIN is an inhibitor of the caspase-related apoptosis pathway. Therefore, the caspase-related pathway was explored by evaluating caspase-3 and PARP activity. As expected, western blot analysis showed that M1 CM increased caspase-3 activation, which triggered PARP cleavage. Such PARP activation was shown in M1 CM-treated cell lysates. However, the findings with M2 CM were opposite to those of M0 CM (Fig. 1d). Flow cytometry results confirmed that M1 CM-induced apoptosis, whereas M2 CM inhibited apoptosis in both HCT116 and HT29 cells (Fig. 1e). The results of the colony formation assay showed that M2 CM significantly increased the proliferation of HT29 cells compared with that of M0 CM, whereas M1 CM reduced proliferation (Fig. 1f). These results suggest that M1 CM increases apoptosis, whereas M2 CM inhibits apoptosis compared with M0 CM in HCT116 and HT29 cells.

### M2 macrophages promote tumor growth of colon cancer cells in vivo

**Fig. 2:**
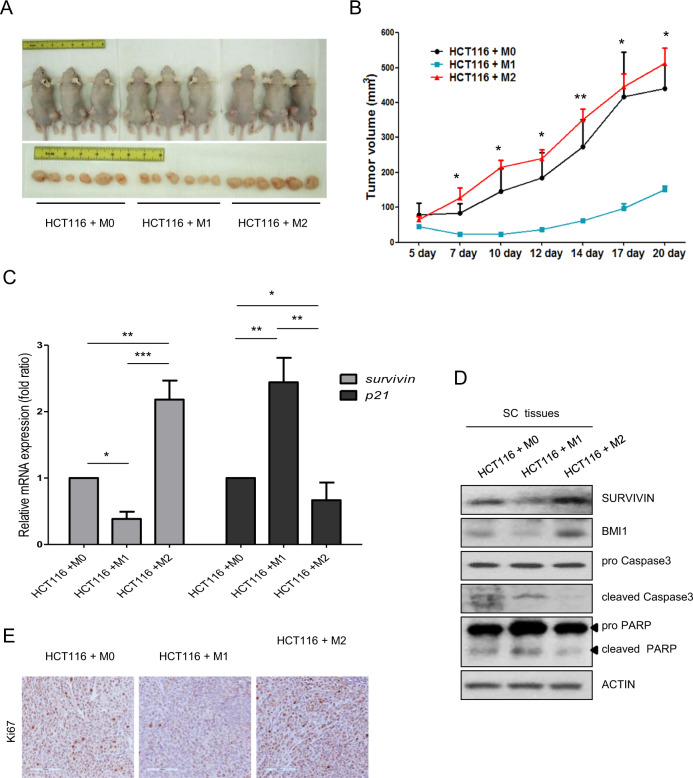
Opposite effects of M1 and M2 macrophages on tumor growth. **a** Tumor images and **b** tumor growth 3 weeks after transplantation of HCT116 cells after long-term coculture with differentiated M0, M1, or M2 macrophages (see Materials and Methods). Tumor size was measured 2–3 times a week with a caliper. Tumor volume was calculated using the following formula: tumor volume = (short length × long length × width)/2. The expression of *SURVIVIN* or *p21* **c**) and proliferation markers **d** in mouse subcutaneous tissues was evaluated by real-time PCR and western blotting, respectively. **e** Ki67 staining (1:200) of mouse subcutaneous tissues three weeks after subcutaneous injection. Scale bar: 100 μm. The results shown are presented as the mean ± SD from three mice per group. Actin was used as a loading control. *P < 0.05; **P < 0.01; ***P < 0.001.

Our current data revealed that M1 CM and M2 CM had opposite roles in the proliferation of colon cancer cells in vitro. To confirm these in vitro findings, we evaluated whether M1 and M2 macrophages had opposite effects on colon cancer cells in vivo using a mouse xenograft model. Before transplantation, HCT116 cells were cocultured with macrophages (M0, M1, or M2) for 7 days, followed by incubation with macrophage CM for three days. Consistent with our in vitro results, the in vivo data also showed that the M1 macrophage coculture condition significantly inhibited tumor growth compared with that of M0 or M2 macrophage coculture conditions in a mouse xenograft model that was transplanted with HCT116 cells (Fig. 2a, b). M2 macrophage coculture tended to promote tumor growth more than M0 macrophage coculture, although the difference between the two was not statistically significant. To determine whether M1 and M2 macrophages regulate tumor growth by apoptosis, mRNA, and protein expression levels in mouse subcutaneous tissues were determined. M1 macrophage stimuli downregulated the mRNA expression level of the survival-related marker *SURVIVIN* but upregulated the mRNA expression level of the apoptosis-related marker *p21*. On the other hand, M2 macrophage stimuli upregulated *SURVIVIN* expression but decreased *p21* expression (Fig. 2c). Consistent with the mRNA data, the protein expression results also showed that M1 macrophages inhibited tumor growth via caspase-mediated apoptosis (Fig. 2d). Next, IHC staining was performed using Ki67, a well-known proliferation marker used in many cancer tissues. IHC results of Ki67 expression showed that the proliferation of M0/M2 macrophage-cocultured HCT116 cells was higher than that of M1 macrophage-cocultured HCT116 cells. In addition, the proliferation of M2 macrophage-cocultured HCT116 cells was higher than that of M0 macrophages (Fig. 2e).

### M2 macrophage CM enhances colon cancer cell migration through epithelial–mesenchymal transition (EMT) regulation

**Fig. 3:**
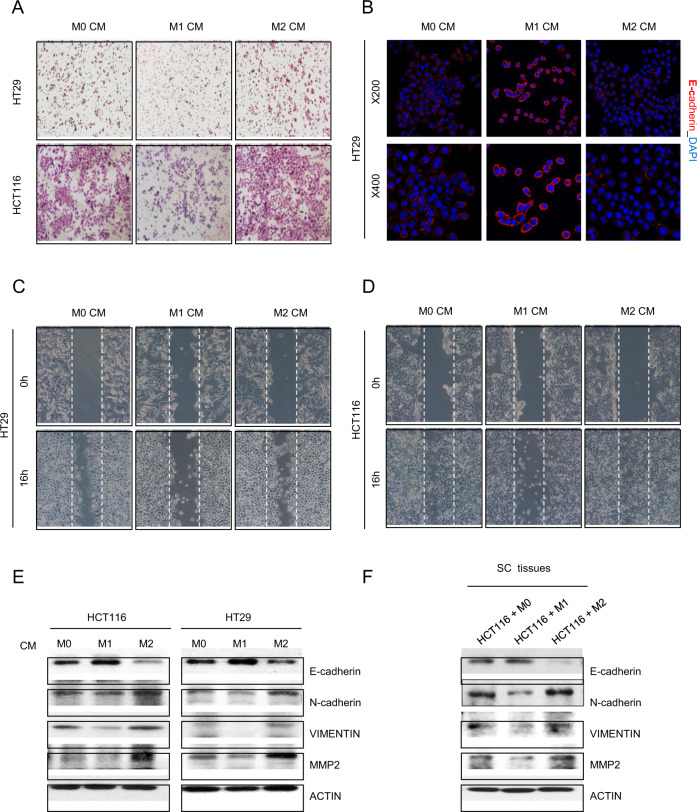
Effects of macrophage CM on EMT in colon cancer cells. **a** Transwell Matrigel invasion assays of HT29 and HCT116 cells after coculture with macrophage CM for 24 h. **b** HT29 cells were subjected to immunofluorescence staining with an antibody against E-cadherin, were mounted with DAPI-containing mounting solution and observed at ×200 and ×400 magnification. A wound-healing assay was performed by creating a wound on a confluent monolayer of HT29 **c** and HCT116 **d** cells during incubation with macrophage CM for 16 h. Expression levels of EMT markers in HCT116 and HT29 cells treated with macrophage CM for 24 h **e** and in mouse subcutaneous tissues **f** were evaluated by western blotting. Actin was used as a loading control.

To assess whether M2 CM induces the EMT in colon cancer cells, invasion and wound-healing assays were conducted using HCT116 and HT29 cells after 24 h of treatment with each macrophage CM. Both the invasion and migration abilities of M2 CM-treated HCT116 and HT29 cells were increased compared with those of cells treated with M0 CM. On the other hand, M1 CM treatment markedly blocked invasion (Fig. 3a) and migration (Fig. 3c, d) of HCT116 and HT29 cells. Loss of membranous E-cadherin expression enables cancer cell invasion^30^. Therefore, the surface expression of E-cadherin in HT29 cells was analyzed using immunofluorescence staining. As anticipated, E-cadherin expression was decreased in M2 CM-treated cells. However, M1 CM obviously induced E-cadherin expression on the membrane of HT29 cells (Fig. 3b). Protein expression of EMT markers in HCT116 and HT29 cells were also determined by western blotting. E-cadherin expression was increased in M1 CM-treated cells. However, M2 CM impeded E-cadherin expression in comparison with that of M0 CM. Other EMT markers, such as N-cadherin, VIMENTIN, and MMP2, also showed similar features (i.e., M1 CM reduced EMT, while M2 CM-induced EMT in parallel lysates, Fig. 3e). Similar results were observed when the expression level of EMT markers was measured in a mouse xenograft model transplanted with HCT116 cells that were cocultured with different macrophage subtypes (Fig. 3f). These results demonstrate that M2 macrophages induce the EMT phenotype in colon cancer cells.

### The FBW7-MCL-1 axis is involved in M1/M2 macrophage-related features of colon cancer cells

**Fig. 4:**
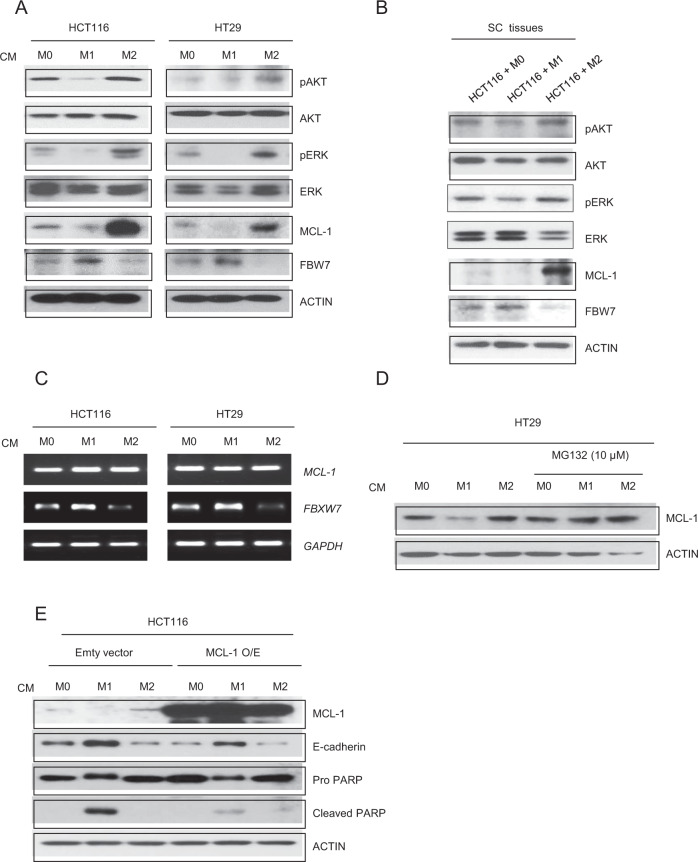
Opposite effects of M1 macrophage CM and M2 macrophage CM on FBW7-mediated MCL-1 degradation of colon cancer cells. Expression levels of AKT, ERK, MCL-1, and FBW7 in **a** macrophage CM-treated HCT116 and HT29 cells and in **b** mouse subcutaneous tissues transplanted with long-term coculture and differentiated M0, M1, and M2 macrophages. **c** mRNA levels of MCL-1 and FBW7 were evaluated by RT-PCR after macrophage CM treatment for 24 h. **d** HT29 cells were exposed to MG132 for 1 h and incubated with macrophage CM for 24 h. **e** HCT116 cells were transfected with either empty vector or MCL-1 expression vector for 24 h, followed by incubation with macrophage CM for another 24 h. Actin was used as a loading control.

The pathways responsible for TAM-induced colon cancer cell proliferation and EMT behavior in HCT116 and HT29 cells, as well as previously obtained mouse subcutaneous tissues, were then determined. MCL-1 is related to both proliferation and the EMT^31,32^. Therefore, MCL-1, as a central regulator of these features, was examined in colon cancer cells that were cocultured with macrophages. It has been reported that AKT and ERK enhance the stability of MCL-1^33,34^. Thus, AKT and ERK activities were evaluated by measuring their phosphorylation in macrophage CM-treated colon cancer cells (Fig. 4a) and previously obtained mouse subcutaneous tumors that arose from implanted HCT116 cells that had been cocultured with macrophages (Fig. 4b). The results showed that in HCT116 and HT29 cells and mouse subcutaneous tumors, coculture with M2 macrophages increased AKT and ERK activation, whereas coculture with M1 macrophages reduced their activation compared with coculture with M0 macrophages. The effects of each macrophage phenotype on MCL-1 protein expression were then examined in parallel cell lysates and tumor lysates. Our results revealed that M1 macrophages markedly decreased MCL-1 protein levels. However, coculture with M2 macrophages enhanced MCL-1 protein expression. Previous studies suggested that F-box/WD repeat-containing protein 7 (FBW7) regulates MCL-1 expression via ubiquitination and degradation^35^. Therefore, FBW7 expression was examined in the same cell and tumor lysates. Western blotting results also indicated that FBW7 was involved in MCL-1 stabilization in colon cancer cells (Fig. 4a) and mouse subcutaneous tumors (Fig. 4b) that were cocultured with macrophages. MCL-1 is also regulated at the transcriptional level. Therefore, RT-PCR was conducted to evaluate the gene expression of *MCL-1* under three CM stimulation conditions. RT-PCR results showed that macrophage subtypes had no effect on MCL-1 mRNA expression. FBW7 mRNA expression was similar to its protein expression pattern. FBW7 expression was relatively high in M1 CM-stimulated cells but low in M2 CM-stimulated cells in comparison with that in M0 CM-treated cells (Fig. 4c). To further confirm that MCL-1 was degraded by the ubiquitin-proteasome pathway, MCL-1 expression in macrophage CM-treated HT29 cells that were pretreated with or without a proteasome inhibitor (MG132) was then examined using western blotting. As shown in Fig. 4d, MG132 restored the protein level of MCL-1 in M1 CM-treated HT29 cells. We next examined whether MCL-1 overexpression (Supplementary Fig. 2B) could reverse the effect of macrophage CM in HCT116 cells. MCL-1 overexpression reduced E-cadherin expression after MCL-1-overexpressing HCT116 cells were incubated with M1 CM. Cleaved PARP expression was decreased in each macrophage CM-treated MCL-1-overexpressing HCT116 cell line compared with that of control empty vector-transfected HCT116 cells (Fig. 4e). Furthermore, by using S64846, an MCL-1-specific inhibitor, we found that MCL-1 inhibition in M2 CM-treated cancer cells increased apoptosis and decreased EMT marker expression (Supplementary Fig. 3A). Next, we conducted a cytokine array to discover which cytokines were affected the most by each macrophage CM. Our results showed that M1 CM contained higher levels of the proinflammatory cytokines CXCL11 and IL-1β than M2 CM, whereas M2 CM had higher levels of the antiinflammatory cytokines CCL5 and IL-8 than M1 CM (Supplementary Fig. 3B). IFN-γ, IL-13, and IL-4 were excluded from analysis because they were used for macrophage polarization.

Our results verified that posttranslational modulation of the FBW7-Mcl-1 axis was an underlying mechanism involved in macrophage-mediated colon cancer cell proliferation and EMT behavior via the activation of AKT and ERK.

### Ex vivo experiments confirm the role of M1 and M2 macrophages in colon cancer cell pathology

**Fig. 5:**
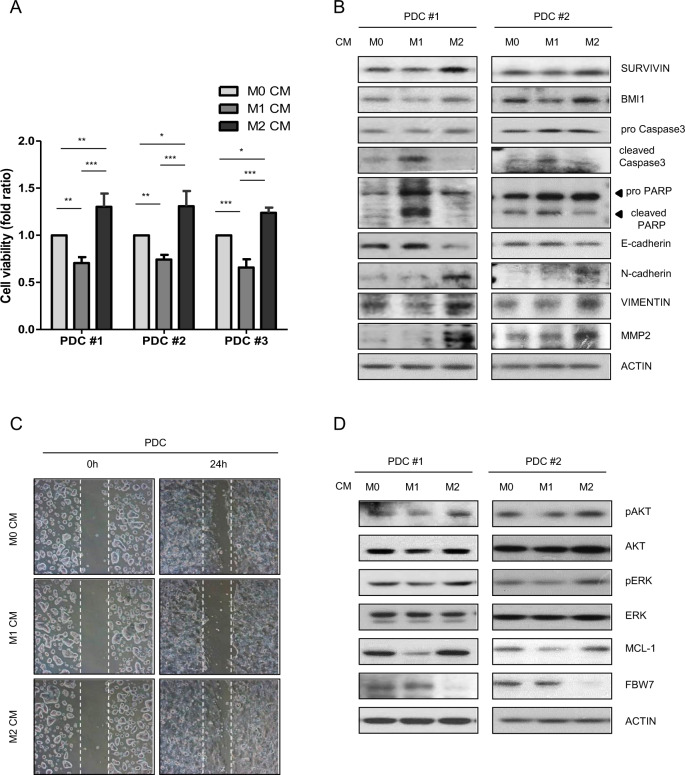
Ex vivo analysis of the role of macrophage CM in colon cancer patient-derived cells (PDCs). **a** Estimation of PDC viability after coculture with macrophage CM for 24 h using the WST-1 assay. Error bars are derived from three independent experiments. **b** Expression levels of apoptosis and EMT markers in macrophage CM-treated PDCs were measured by western blotting. **c** Wound-healing ability was evaluated by creating wounds on a confluent monolayer of PDCs using 1-Dish 35-mm-high culture inserts. **d** Activation of AKT and ERK and expression levels of MCL-1 and FBW7 assessed by western blotting. Actin was used as a loading control. The results are presented as the mean ± SE. **P < 0.01; ***P < 0.001.

To further confirm the data obtained above, ex vivo analysis was performed using PDCs (Supplementary Fig. 1E). M1 CM significantly decreased the viability of PDCs, whereas M2 CM significantly increased the viability of PDCs compared to that of M0 CM treatment (Fig. 5a). To determine whether cell survival was associated with macrophage CM, western blotting was performed using antibodies against representative apoptosis markers. As expected, M1 CM-induced cell death through apoptosis, whereas M2 CM enhanced survival of PDCs (Fig. 5b). The effect of macrophage CM on the EMT features of PDCs was also determined using the same respective cell lysates. The expression levels of the well-known EMT markers E-cadherin, N-cadherin, VIMENTIN, and MMP2 in M0, M1, or M2 CM-treated PDCs were also determined. The results confirmed that M2 macrophages enhanced EMT progression (Fig. 5b). The representative image shown in Fig. 5c revealed that M1 CM inhibited the migration of PDCs, whereas M2 CM-induced migration of PDCs compared with that of M0 CM. Consistent with our previous results (Fig. 4) showing that posttranslational modulation of the FBW7-Mcl-1 axis was an underlying mechanism involved in macrophage-mediated colon cancer cell proliferation, the involvement of the FBW7-Mcl-1 axis in PDCs was also found in CM-treated macrophages (Fig. 5d).

### Effects of macrophage PI3Kγ inhibition on colon cancer cell viability and EMT status

**Fig. 6:**
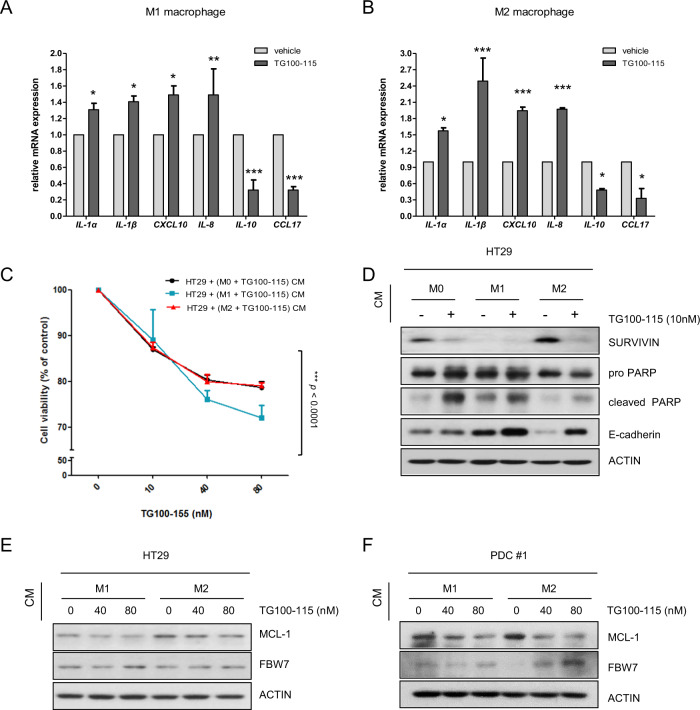
Role of PI3Kγ in TAM-mediated colon cancer cell viability and EMT characteristics. mRNA expression levels of genes involved in the proinflammatory response (*IL-1α, IL-1β, CXCL10*, and *IL-8*) and antiinflammatory response (*IL-10* and *CCL17*) were evaluated by real-time PCR in M1 **a** and M2 **b** macrophages with or without treatment with 10 nm TG100-115 (PI3Kγ inhibitor) for 18 h. **c** HT29 cells were exposed to TG100-115-treated macrophage CM for 24 h. Cell viability was then measured by WST-1 assay. **d** Expression levels of apoptosis- and EMT-related markers in CM-treated HT29 cells. CMs were derived from TG100-115 (10 nm)-treated or untreated M0, M1, and M2 macrophages. HT29 cells **e** and PDCs **f** were incubated with TG100-115-treated macrophage CM for 24 h. Protein levels of MCL-1 and FBW7 were evaluated using western blotting. Actin was used as a loading control. The results are presented as the mean ± SE. *P < 0.05; **P < 0.01; ***P < 0.001.

Although our results confirmed the effect of macrophage CM on colon cancer cell survival and EMT phenotypes, target macrophage molecules need to be determined to apply these findings to clinical immunotherapy. PI3Kγ is specifically expressed in myeloid cells but not in cancer cells. PI3Kγ enhances myeloid cell trafficking during the inflammatory response and carcinogenesis^36^. Hence, PI3Kγ was a focus of this study as a promising candidate target for colon cancer immune therapy. As small-molecule inhibitors might be more useful for clinical approaches than genetic modulation, the biological effects of the PI3Kγ inhibitor TG100-115 (Selleckchem) were determined. RT-PCR performed in triplicate revealed that proinflammatory cytokines (upper panel) were upregulated in M1 macrophages, whereas antiinflammatory cytokines (lower panel) were upregulated in M2 macrophages (Supplementary Fig. 1C). TG100-115-induced PI3Kγ inhibition significantly stimulated proinflammatory cytokine expression and M1 macrophage marker expression but reduced antiinflammatory cytokine expression and M2 macrophage marker expression in both M1 and M2-differentiated macrophages (Fig. 6a, b, Supplementary Fig. 2C). Similar results were observed when M1 and M2 macrophages were treated with another well-known PI3Kγ inhibitor, IPI-549 (Supplementary Fig. 3C). In other words, PI3Kγ controls macrophage switching between immune tolerance and immune surveillance by regulating cytokines. Next, CM was collected from TG100-115-treated M0, M1, or M2 macrophages. As expected, M0, M1, and M2 CM collected from TG100-115-treated macrophages significantly reduced the viability of HT29 cells (Fig. 6c) and PDCs (Supplementary Fig. 2D) in a dose-dependent manner regardless of macrophage subtype. These results suggest that blocking PI3Kγ in macrophages promotes cytotoxic effects in colon cancer cells. Subsequently, the effect of PI3Kγ inhibition in macrophages on EMT and the apoptosis-related death of colon cancer cells was examined by western blotting. Our results showed that M0, M1, and M2 CM from TG100-115-treated macrophages restored the protein levels of E-cadherin (an EMT marker) as well as the apoptosis markers cleaved PARP and SURVIVIN in HT29 cells regardless of macrophage subtype (Fig. 6d). Furthermore, CM from TG100-115-treated macrophages at the tested concentrations reduced MCL-1 protein expression but enhanced FBW7 expression in both HT29 cells (Fig. 6e) and PDCs (Fig. 6f) regardless of macrophage subtype. The WST-1 assay showed that TG100-115 had no effect on the viability of M1 macrophages, whereas TG100-115 significantly decreased the viability of M2 macrophages in a dose-dependent manner (Supplementary Fig. 2E). Collectively, these data indicate that PI3Kγ targeting in TAMs could switch macrophage-derived cytokines, thus affecting colon cancer cell growth and EMT features.

### Inhibition of PI3Kγ attenuates tumor growth in xenograft models associated with infiltrated macrophages

**Fig. 7:**
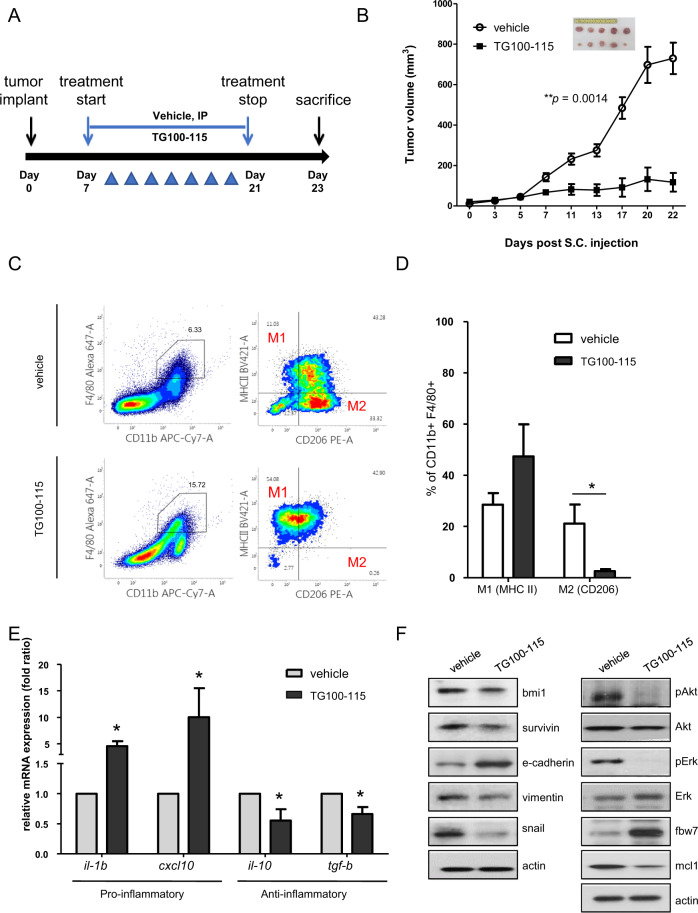
Inhibition of PI3Kγ attenuates tumor growth in xenograft models associated with infiltrated macrophages. **a** Experimental procedure illustrating the TG100-115 treatment regimen in BALB/c mice. **b** CT26 cells (5 × 10^5^ cells/mouse) were subcutaneously injected into the flanks of 6-week-old mice. Mean tumor volume of subcutaneously implanted vehicle- or TG100-115-treated mice (*n* = 5) and representative images of subcutaneous tumors at day 16 after treatment with vehicle or TG100-115 (box) are shown. **c** FACS analysis and quantification of CD11b^+^ F4/80^+^ (TAM) cell populations in CT26 tumors at day 14 posttreatment (*n* = 5) and expression levels of MHCII (M1) and CD206 (M2) in CD11b^+^ F4/80^+^ cell populations. **d** Graph showing the percentage of each population (M1, M2) in the vehicle-treated group in comparison with the TG100-115-treated group. **e** mRNA expression levels of genes involved in the proinflammatory response (*il-1β, cxcl10*) and antiinflammatory response (*il-10* and *tgf-β*) were evaluated by real-time PCR in vehicle and TG100-155-treated groups. **f** Representative western blot analysis showing survival/EMT-related protein expression as well as ERK/AKT-FBW7-MCL-1 signal axis regulation in vehicle- and TG100-155-treated mouse tumor tissues.

To verify our results, we used a syngeneic mouse model of BALB/c wild-type mice and the CT26 mouse colon cancer cell line. In brief, 5 × 10^5^ cells were subcutaneously injected into the mouse flank, and TG100-115 was used for treatment every other day via intraperitoneal injection (Fig. 7a). The mice treated with the PI3Kγ antagonist TG100-115 showed dramatically suppressed tumor growth in the CT26 xenograft model (Fig. 7b). To observe macrophage infiltration in each tissue, we dissociated tumor cells and performed cytometry analysis using specific markers for total macrophages (CD11b^+^F4/80^+^). We found that PI3Kγ inhibition induced macrophage infiltration into the tumor region (Fig. 7c left panel). Blocking PI3Kγ activity with TG100-115 treatment increased the proportion of M1-like macrophages (CD11b^+^F4/80^+^MHC II^+^) but markedly reduced M2-like macrophages (CD11b^+^F4/80^+^CD206^+^) (Fig. 7c right panel, Fig. 7d). Relative mRNA expression levels of M1-like proinflammatory cytokines (*il-1β* and *cxcl10*) were upregulated, whereas those of M2-like antiinflammatory cytokines (*il-10* and *tgf-β*) were downregulated in TG100-115-treated tumors (Fig. 7e). Moreover, the expression levels of survival markers and EMT markers in TG100-115-treated mouse tissues confirmed that PI3Kγ inhibition reversed tumor progression through the FBW7-MCL-1 axis (Fig. 7f).

### Association between M1 and M2 macrophage infiltration and prognostic importance

**Fig. 8:**
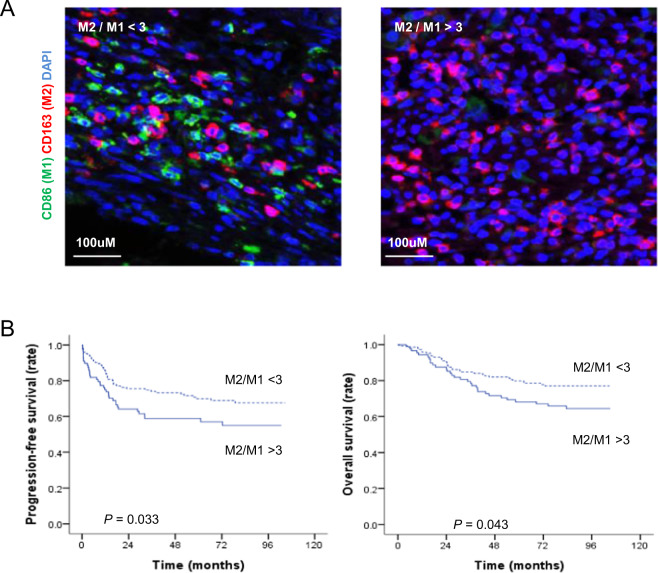
Survival analysis according to the M2 marker (CD163) to M1 marker (CD86) ratio in the validation cohort. **a** Representative immunohistochemical staining for markers of M1 macrophages (CD86, green) and M2 macrophages (CD163, red) in CRC tissues. Dried slides were scanned using the PerkinElmer Vectra 3.0 platform at ×20 magnification. Nuclei were shown by DAPI staining (blue). Scale bar: 100 µm. **b** Kaplan–Meier curves for progression-free survival (left panel) and overall survival (right panel) of the two groups were divided by an optimal cutoff value of 3 for the ratio of M2 marker (CD163) to M1 marker (CD86). Statistical analysis by long-rank test: *p* = 0.033 and *p* = 0.043, respectively.

The major M1 macrophage biomarker CD86 and M2 macrophage biomarker CD163^37^ were used to identify TAM phenotypes. To test whether CD86 and CD163 could be used to distinguish different subpopulations of M1 or M2 macrophages, CRC tissues were analyzed by multiplex staining using the Opal Kit (see Materials and Methods). The results showed that CD86 and CD163 were expressed in different populations of macrophages. The amounts of M2 (CD163^+^) and M1 (CD86^+^) macrophages showed individual variation in the tumor microenvironment (Fig. 8a). To determine the prognostic impact of M1 or M2 macrophage infiltration in the tumor microenvironment, survival outcomes were analyzed with Kaplan–Meier analysis among a total of 232 CRC patients (stage I, *n* = 32; stage II, *n* = 70; stage III, *n* = 76; and stage IV, *n* = 54). Based on the results of the M1 and M2 macrophage markers, these 232 cases were divided into the following two groups: (1) M2/M1 < 3 and (2) M2/M1 > 3. The results revealed that patients with M2/M1 < 3 had significantly better PFS (*p* = 0.033, Fig. 8b, left panel) and overall survival (*p* = 0.043, Fig. 8b, right panel) than patients with M2/M1 > 3.

## Discussion

Multiple studies have shown that TAMs are associated with cancer progression in many types of malignancies, including liver, breast, gastric, and lung cancers^8,38,39^. Although these studies have implicated infiltrated TAM density and polarization status in 5-year survival outcomes, the impact of the ratio of infiltrated TAMs on survival in CRC remains controversial^16,40^. Moreover, mechanistic studies of TAM-related tumor progression and potential immunotherapeutic targets in TAMs have been sparse. To the best of our knowledge, this is the first attempt to estimate the mechanistic role of M1/M2 TAM subtypes in MCL-1-mediated cancer cell proliferation and the EMT process using in vivo, in vitro, and ex vivo approaches. In addition, the effects of inhibiting macrophage PI3Kγ on tumor suppression were examined in this study. Our results could help validate the immunotherapeutic relevance of targeting PI3Kγ in CRC treatment. The results of the clinical data analysis of 232 CRC patients revealed that the ratio of M2 to M1 macrophages was associated with the survival outcomes of CRC patients (Fig. 8). Our results clearly showed that PFS and overall survival were significantly prolonged in patients with an M2 to M1 macrophage ratio <3 compared with those with a ratio larger than 3. In contrast to our results, Nagorsen et al.^41^ reported that M2 macrophage infiltration in CRC is correlated with better survival outcomes. However, their study did not estimate the parallel presence of M1 macrophage infiltration. The distribution of M1 and M2 macrophages and their ratio in CRC are believed to be very important in developing comprehensive immunotherapeutic strategies. Similar to our results, Edin et al.^16^ found a positive correlation between infiltrated M1 macrophages and survival outcomes in CRC patients. However, unlike our results, they concluded that M2 macrophage infiltration was increased concomitantly with M1 macrophage infiltration. The unique value of our clinical analysis compared to previous reports was that we revealed that the M2/M1 ratio could predict better prognostic outcomes. Both in vitro and in vivo studies demonstrated that M1 and M2 macrophages had opposite effects on the proliferation and EMT phenotypes of colon cancer cells. Data from colon cancer cell lines and a mouse xenograft model consistently showed that the AKT and ERK pathways were associated with FBW7-related MCL-1 degradation in M1 or M2 cocultured colon cancer cells. To the best of our knowledge, this is the first study to use an ex vivo approach with PDCs to confirm our in vitro and in vivo data. PDCs are generated by directly resecting patient tumors into single cells. Such models can replicate the features of primary patient tumors. This can help overcome potential barriers of in vitro models^42^. Hence, our study went one step further than existing reports on the role of TAMs in the colon cancer tumor microenvironment. Intracellular PI3Kγ has recently been suggested to be a target for immunotherapy in macrophages. PI3Kγ inhibition can switch macrophage phenotypes from immune tolerance to immune surveillance by regulating the NFκB and mTOR-C/EBP pathways^26,43^. In agreement with previous reports, our results revealed that PI3Kγ inhibition increased antitumor cytokines and decreased protumor cytokines in macrophages, thus enhancing apoptosis-related cancer cell death regardless of the TAM subtype. Moreover, apoptosis marker and EMT marker expression, as well as the FBW7-MCL-1 axis, were reversed via CM obtained from TG110-115-treated M1 and M2 macrophages. Furthermore, PI3Kγ inhibition demonstrated potential therapeutic use in an in vivo xenograft model, as regression was seen in 80% of syngeneic mouse tumors after treatment with TG100-115. We found that PI3Kγ inhibition increased the number of tumor-infiltrated macrophages and switched their polarization from immunosuppressive M2 macrophages to more proinflammatory M1 macrophages. Furthermore, we confirmed that tumor regression was related to FBW7-MCL-1 signaling associated with the polarization of infiltrated macrophages. Moreover, TG100-115 significantly decreased the viability of M2 macrophages without significantly affecting the viability of M1 macrophages. These results indicate that PI3Kγ in macrophages might be a promising target in the development of immunotherapy for colon cancer.

Since one study reported a 0% response rate of colon cancer MSS patients to pembrolizumab in 2015^44^, the results of PD-1/PD-L1 antibody treatment for MSS tumor types have not been established. Immune checkpoint inhibitor treatment for colon cancer patients has only been established in MSI patients, who represent <15% of colon cancer patients^45,46^. MSI patients exhibit microsatellite-unstable disease types that are characterized as mismatch repair deficient. Unlike the MSS tumor type, which is a mismatch repair proficient disease type, MSI tumors contain many mutations that create unique new antigens. Hence, active immunotherapy strategies that involve macrophage targeting for MSS tumor types are promising. Hence, the results we present here provide a rationale for targeting macrophages by reprogramming macrophage characteristics from protumoral to antitumoral, which may affect the colon tumor microenvironment from cold tumors to hot tumors.

In summary, the correlation between the M1 to M2 ratio and survival outcomes and the underlying signaling pathway involved were examined by using in vitro, in vivo, and ex vivo approaches in this study. Our results demonstrated that the cancer cell progression induced by M2 macrophages was mechanistically linked to FBW7-mediated MCL-1 stabilization in colon cancer cells. Moreover, inhibition of endogenous PI3Kγ in M2 macrophages could switch macrophage function from “protumor” to “antitumor”, thus affecting colon cancer cell survival in the tumor microenvironment. Although further improvements are required to clarify the internal signaling pathway of PI3Kγ in TAMs, our results identified FBW7-MCL-1 as a key axis in M2 macrophage-related colon cancer cell progression.

## Supplementary information

supplementary information
