# Supplementary material for: The FBW7-MCL-1 axis is key in M1 and M2 macrophage-related colon cancer cell progression: validating the immunotherapeutic value of targeting PI3Kγ
Source: Exp Mol Med. 2020 May 22;52(5):815–31. doi: 10.1038/s12276-020-0436-7 (PMC7272616; doi:10.1038/s12276-020-0436-7)
Supplement: Supplementary file 1 — supplementary information [file 12276_2020_436_MOESM1_ESM.doc]

**
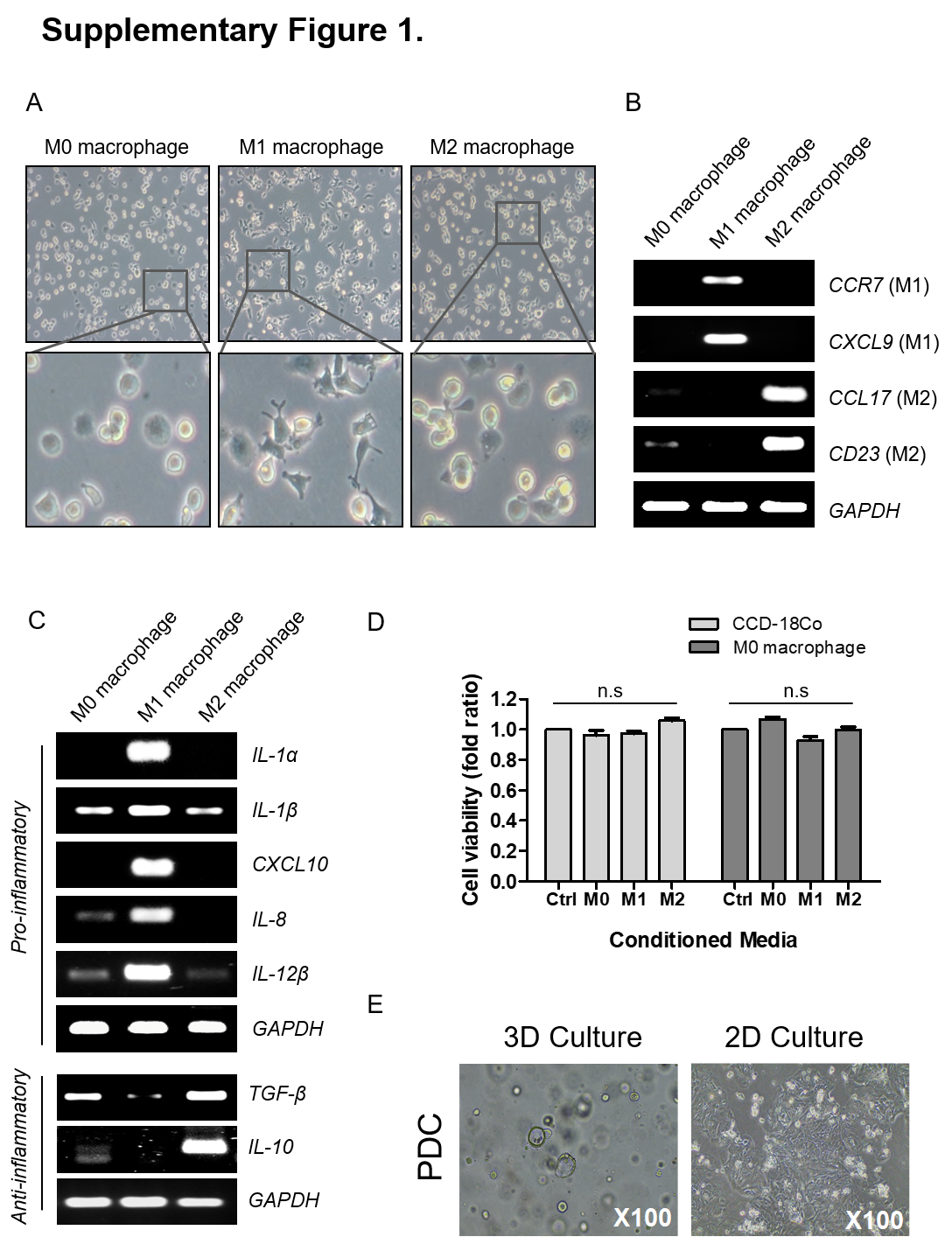
**

**
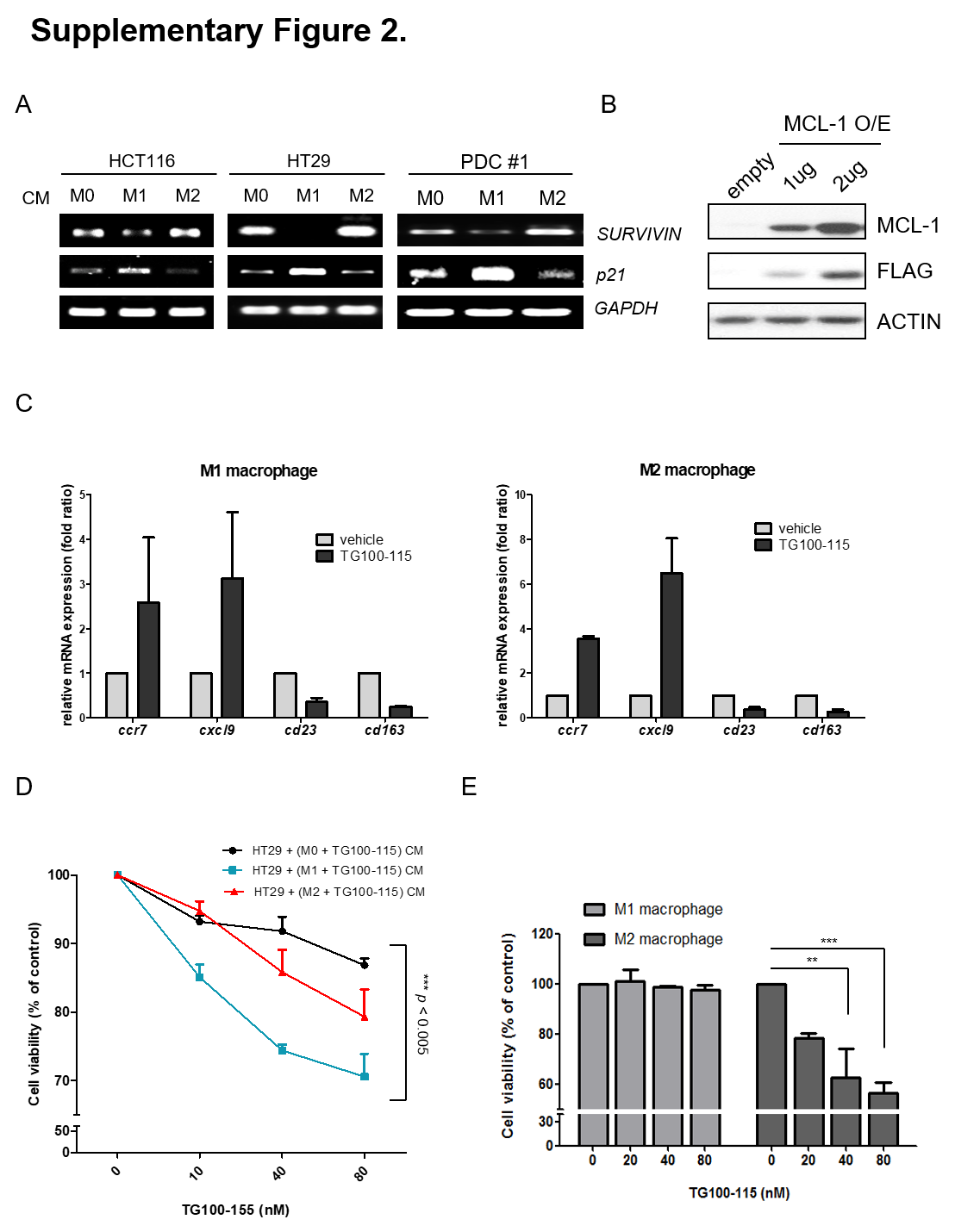
**

**
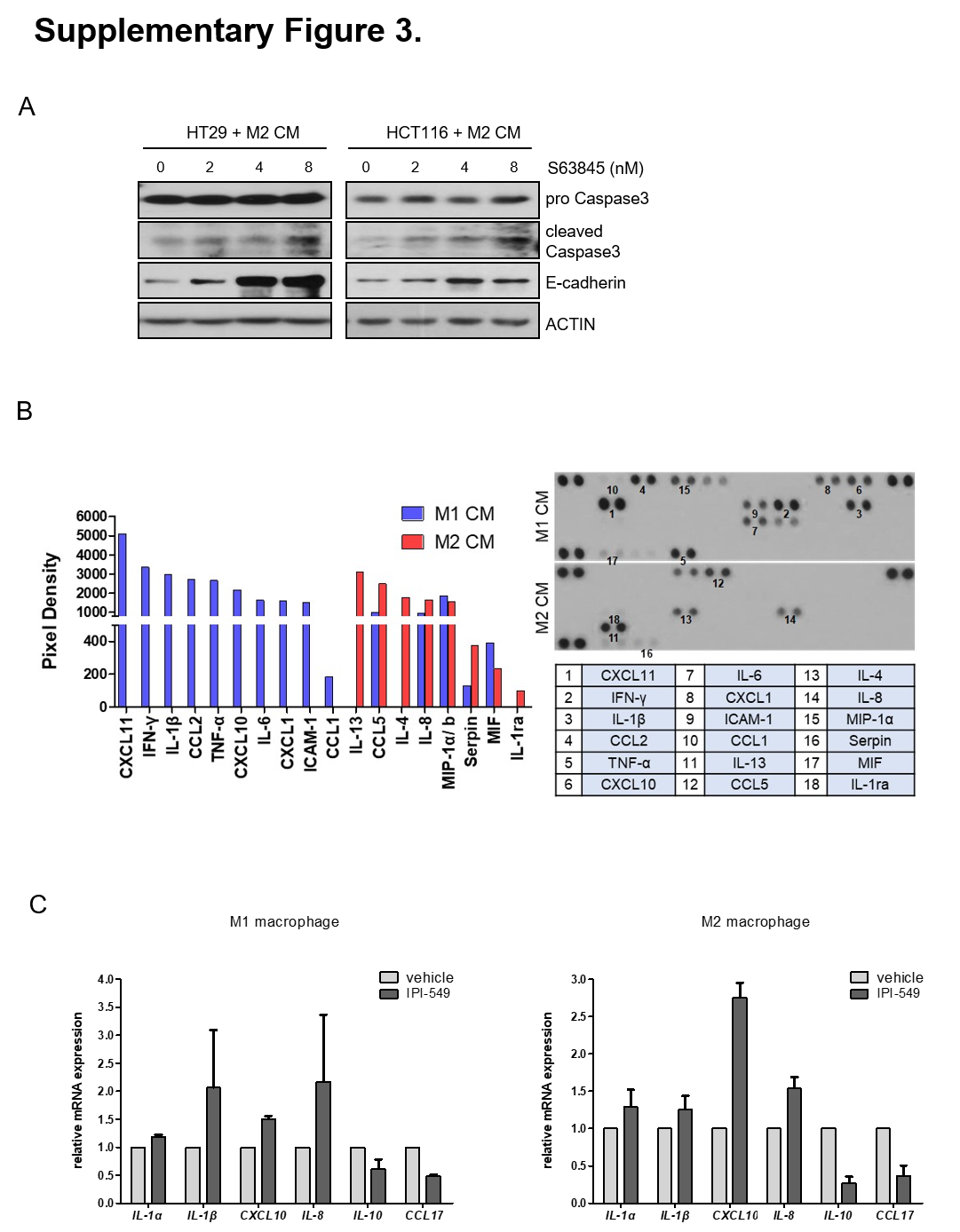
**


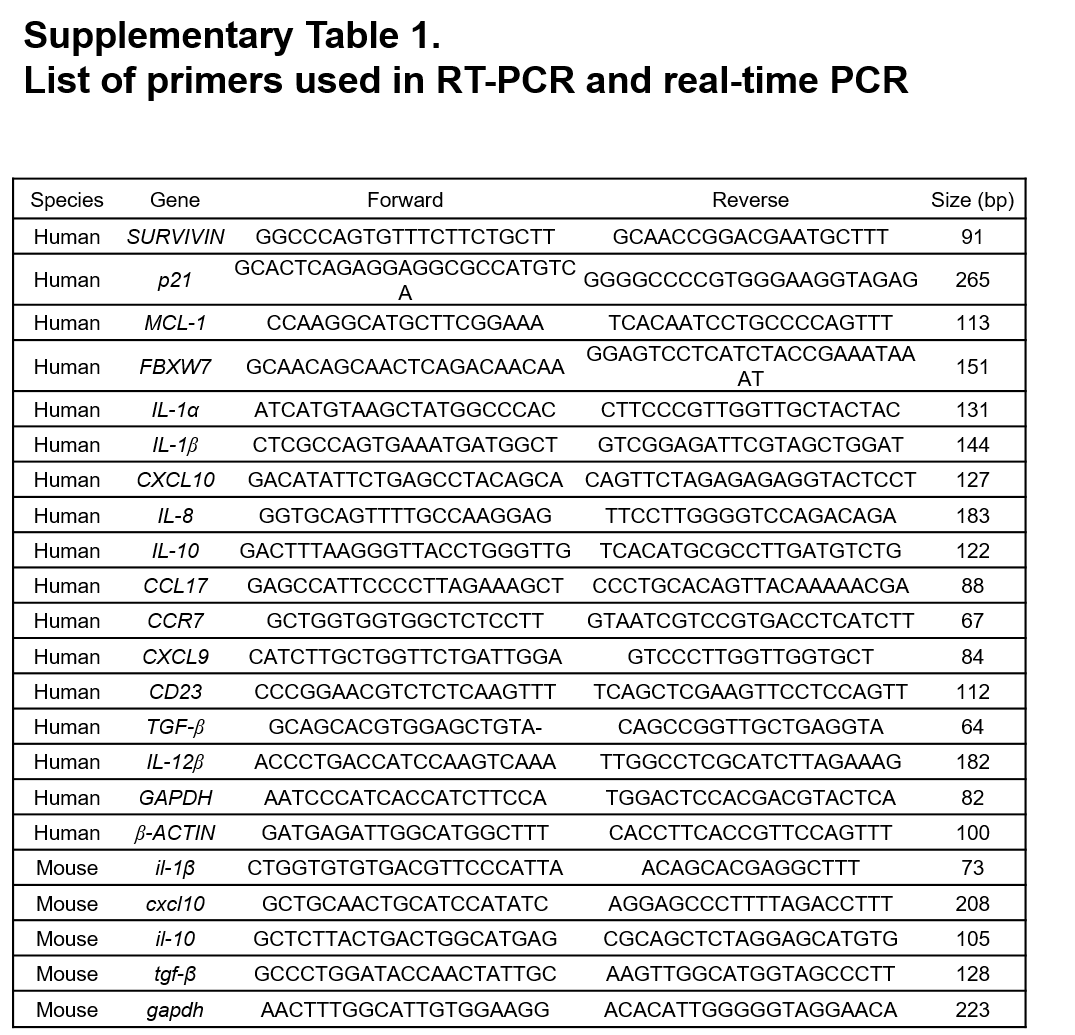


**Supplementary Figure Legends**

**Supplementary Figure 1. Differentiation of THP-1 into M0, M1, and M2 macrophages.**

(A) Phenotypic changes in differentiated THP-1 cells in response to standard stimuli (see Materials and Methods). Representative images of cells taken at x200 magnification (upper panel) and digital zoom (lower panel). (B) RT-PCR analysis showing expression levels of M1 and M2 macrophage polarized markers in differentiated THP-1 cells. (C) mRNA expression levels of pro-inflammatory cytokines (*IL-1α, IL-1β, CXCL10, IL-8, IL-12β*) and anti-inflammatory cytokines (*TGF-β, IL-10*) in differentiated macrophages measured by RT-PCR. GAPDH was used as a loading control. (D) Estimation of both CCD-18Co and M0 macrophage viability after co-culture with macrophage CM for 24 hours using WST-1 assay. (E) Representative images of 3D and 2D cultured patient derived cells (PDC): x 100 magnifications.

**Supplementary Figure 2. Effect of macrophage conditioned medium (CM) on the viability of colon cancer cells and patient-derived cells (PDCs).**

(A) HCT116 cells, HT29 cells, and PDC #1 were treated with macrophage CM for 24 hours and expression levels of apoptosis markers *SURVIVIN* and *p21* were measured by RT-PCR. *GAPDH* was used as a loading control. (B) Total cell lysates were subjected to western blot analysis to confirm MCL-1 overexpression. (C) mRNA expression levels of M1 markers (*ccr7, cxcl9*) and M2 markers (*cd23, ccl17*) were evaluated by real-time PCR in M1 (left) and M2 (right) macrophages with or without treatment by 10 nM TG100-115. (D) PDC #1 cells were exposed to TG100-115-treated-macrophage CM for 24 hours. (E) Differentiated M1 and M2 macrophages were exposed to TG100-115 at the indicated dose for 24 hours. Cell viability was then measured by WST-1 assay. Results shown are mean values ± SE. ***, *P* < 0.005.

**Supplementary Figure 3. Effect of macrophage conditioned medium (CM) on the viability of colon cancer cells and patient-derived cells (PDCs).**

(A) Expression levels of apoptosis-related marker, Caspase3 and EMT- related marker, E-cadherin in CM treated HT29 and HCT116 cells. CMs were derived from S63845 treated M2 macrophages. (B) Expression of cytokines in macrophage CM using cytokine array (left) and representative image of cytokine dots (right). (C) mRNA expression levels of genes involved in pro-inflammatory response (IL-1α, IL-1β, CXCL10, and IL-8) and anti-inflammatory response (IL-10 and CCL17) were evaluated by real-time PCR in M1 and M2 macrophages with or without treatment by 100 nM IPI-549 (PI3Kγ inhibitor) for 18 hours.
